# The Effect of Simvastatin on Odontoblastic Differentiation of Human Dental Pulp Stem Cells: An In Vitro Study

**DOI:** 10.3390/dj13090428

**Published:** 2025-09-16

**Authors:** Carmela Del Giudice, Flavia Iaculli, Carlo Rengo, Alessandro Salucci, Gianrico Spagnuolo, Francesco Riccitiello, Maurizio Bossù, Antonella Polimeni, Gianni Di Giorgio

**Affiliations:** 1 Department of Neurosciences, Reproductive and Odontostomatological Sciences, University of Naples Federico II, 80131 Naples, Italy; delgiudicecarmela93@gmail.com (C.D.G.); rengocarlo@gmail.com (C.R.); riccitie@unina.it (F.R.); 2 Department of Oral and Maxillofacial Sciences, Sapienza University of Rome, 00161 Rome, Italy; flavia.iaculli@uniroma1.it (F.I.); alessandro.salucci@uniroma1.it (A.S.); maurizio.bossu@uniroma1.it (M.B.); antonella.polimeni@uniroma1.it (A.P.); gianni.digiorgio@uniroma1.it (G.D.G.)

**Keywords:** dental pulp stem cells, dentin–pulp complex, odontoblasts, reparative endodontics, simvastatin

## Abstract

**Objectives:** The aim of the current in vitro study was to assess the effect of simvastatin on the early-stage differentiation of human dental pulp stem cells (hDPSCs) in an odontogenic pattern by evaluating the expression of specific odontogenic-related genes. **Methods:** hDPSCs were cultured in the presence of different concentrations of simvastatin (0.1, 0.5, 1, 5, and 10 µM) to evaluate cytotoxicity. Moreover, osteogenic differentiation was assessed by Alkaline Phosphatase (ALP) activity and alizarin red staining (ARS) after 7 days of culture. Finally, odontogenic-related gene (OCN, MEPE, DSPP, and DMP-1) expression analysis was performed. **Results:** Three days after treatment, higher concentrations of simvastatin (1, 5, and 10 µM) significantly limited cell viability. Upregulation of ALP activity and odontoblastic cell-related genes (OCN and MEPE) was observed in the presence of 1 µM simvastatin. The expression was statistically higher for ALP (*p* = 0.0001) and OCN (*p* = 0.0231). On the other hand, comparable or slightly less effect concerning mineralization ability with respect to the control group, as well as in the expression of DSPP and DMP-1, was observed. **Conclusions:** Simvastatin demonstrated a positive influence on dentinogenesis by improving the expression of specific markers such as MEPE and OCN. However, its effect on inflammation reduction and the potential to be used in combination with other materials should be further assessed. Simvastatin might be successfully applied in the regeneration of damaged dental pulp tissues and promotion of reparative dentinogenesis. Additional studies should be carried out to support the obtained outcomes.

## 1. Introduction

Dental caries and trauma may involve pulp tissue in different ways, potentially impacting tooth development and maintenance of vitality. Pulpitis in primary and permanent teeth can be effectively treated through vital pulp therapy procedures [1,2] promoting the dentinal reparative process [3]. On the other hand, pulpal necrosis hampers the root development of immature dental elements and increases fracture risk [4]. In this light, regenerative approaches to the dentin–pulp complex are recently proposed as valid alternatives for immature tooth treatment [5]. However, the promotion of both reparative and regenerative processes is clinically challenging and strictly depends on the choice of specific materials and procedures. Pulpotomy—considered the therapy of choice in vital pulp therapy (VPT)—yielded encouraging middle-term results both in primary and permanent dental elements, with the aim to preserve pulp vitality and function and to provide a valid alternative to traditional root canal therapy [6,7]. On the contrary, the aim of tooth revascularization—or revitalization—is to induce the restoration of a pulp-like tissue within the root canal by promoting blood influx from the periapical area. Originally introduced as an option for apexification [8,9], the proper regeneration of the dentin–pulp complex might be obtained by the differentiation of stem cells from the apical papilla that move into the root canal system [10,11,12].

Statins, drugs commonly used to treat hyperlipidemia, demonstrated multiple functions, including anti-inflammation properties, the promotion of angiogenesis, and the improvement of vascular endothelial cell function [13]. Al-Natour B et al. reported that statin-class drugs reduced the expression of inflammatory cytokines (i.e., IL8, IL6, CCL21, AQP9, and MMP-9) in an ex vivo pulpitis model [14], suggesting a potential effect on decreasing inflammation in the residual radicular pulp after pulpotomy and improving tissue healing. Moreover, statins positively affect the bone morphogenetic protein (BMP)-2 pathway, enhancing mineralized bone tissue formation by stimulating osteoblasts [15]. Accordingly, simvastatin—a 3-hydroxy-3-methylglutaryl coenzyme A reductase inhibitor—has been shown to promote human dental pulp stem cell (hDPSC) differentiation in an odontogenic pattern, favoring the deposition of mineralized dentin-like tissue after pulpotomy and contributing to the potential regeneration of the dentin–pulp complex in the case of pulp necrosis [13,16]. Although the influence of statins on bone tissue metabolism has been well-documented, the capacity of hDPSCs to differentiate in odontoblastic-like cells remains still controversial. Therefore, the aim of the present in vitro study was to assess the early differentiation of hDPSCs exposed to different concentrations of simvastatin by evaluating the expression of specific odontogenic-related genes. The null hypothesis was that simvastatin has no effect on hDPSC differentiation in odontoblasts.

## 2. Materials and Methods

### 2.1. Human Dental Pulp Stem Cell (hDPSC) Culture

hDPSCs (PT-5025, Lonza, Basel, Switzerland) were cultured in α-minimum essential medium (αMEM) supplemented with 10% fetal bovine serum (FBS, GIBCO Thermo Fisher Scientific, Waltham, MA, USA), 2 mM glutamine, and 1% P/S (Sigma-Aldrich, St. Louis, MO, USA) (Basal Medium, BM) in an incubator at the temperature of 37 °C and 5% CO_2_. hDPSCs from passages 4–7 were used. Cells were cultured with different concentrations of simvastatin (S6196-5MG, Sigma-Aldrich), as described in each experimental design, and untreaded cells represented the control group.

### 2.2. Viability Assay

The evaluation of simvastatin cytotoxicity on hDPSCs was conducted using the MTT [3-(4,5-dimethylthiazol-2-yl)-2,5-diphenyltetrazolium bromide] assay in accordance with the manufacturer’s instructions (Sigma-Aldrich) and as previously described [17]. The cells were seeded into a 96-well plate at an initial density of 5 × 10^3^ cells per well. After 24 h, hDPSCs were treated with simvastatin at concentrations of 0.1, 0.5, 1, 5, and 10 µM, and the 96-well plate was incubated for 72 h [13,18,19,20]. Subsequently, the medium was aspirated by each well and MTT solution (final concentration 5 µg/mL) was added, followed by a 4 h incubation at 37 °C and 5% CO_2_. Upon formation of purple precipitates, 100 µL acidic isopropanol (0.04 N HCl in absolute isopropanol) was added to create the formazan dye soluble. Absorbance was measured at 570 nm using a microplate reader (Tecan, Grödic, Austria). Cells cultured with Dulbecco’s Modified Eagle Medium (DMEM) were used as control. The outcomes were obtained by three independent experiments conducted in triplicate.

### 2.3. Alkaline Phosphatase Activity

The cells were cultured on a 48-well plate at a density of 2.5 × 10^4^ cells/well. After allowing them to adhere overnight, the cells were cultured with osteogenic medium in DMEM, as above, supplemented with 50 µM ascorbic acid, 10 mM β-glycerophosphate, and 100 nM dexamethasone (Sigma-Aldrich) in the presence of simvastatin 0.1, 0.5, and 1 µM for 7 days [21]. After 7 days, Alkaline Phosphatase (ALP) activity was detected using an Alkaline Phosphatase Activity Assay Kit (Elabscience, E-BC-K091, Houston, TX, USA) and calculated as enzymatic units/mg protein. The untreated cells in osteogenic conditions represented the control group.

### 2.4. Alizarin Red Staining Assay

hDPSCs were plated in a 24-well plate at a density of 5 × 10^4^ cells/well, and when cells reached confluence, they were cultured with osteogenic medium, as previously described, with simvastatin 0.1, 0.5, and 1 µM. The treatment was maintained for 7 days to assess early-stage mineralization. The accumulated calcium deposition was detected using alizarin red staining solution as previously reported [22]. In brief, the cells were fixed with 4% paraformaldehyde (Sigma-Aldrich) for 15 min at room temperature, then the fixative was removed and the cells were washed with distilled water. Then, 250 µL of alizarin red staining solution (Merck, TMS-008-C, Rahway, NJ, USA) was added to the cells for at least 30 min. After the cells were washed two times with deionized water, photographs were obtained by means of an optical microscope equipped with a digital camera at 10× magnification (LEICA DMi6000, Leica Microsystems Wetzlar GmbH, Wetzlar, Germany). The quantification of alizarin red was obtained by adding 10% acetic acid to cell samples for 30 min while shaking and then the samples were quantified by measuring the absorbance at a wavelength of 405 nm using a microplate reader (Tecan, Grödic, Austria) [23].

### 2.5. Odontogenic-Related Gene Expression Analysis

The cells were plated in a 48-well plate at a density of 2.5 × 10^4^ cells/well and, when the cells reached confluence, they were cultured with osteogenic medium and exposed to simvastatin 0.1, 0.5, and 1 µM for 7 days. hDPSCs in osteogenic medium without simvastatin were used as the control group. To establish the expression levels of odontogenic-related genes, total RNA was obtained from cell cultures using the TriZOL Reagent (Invitrogen), according to the manufacturer. Reverse transcription was performed using 1.0 μg total RNA and a High Capacity cDNA Reverse Transcription Kit (Applied Biosystems, Waltham, MA, USA). The resulting cDNA was amplified by real-time polymerase chain reaction (PCR) by means of Power SYBR Green PCR Master Mix (Applied Biosystems) and specific primers for osteocalcin (OCN), matrix extracellular phosphoglycoprotein (MEPE), dentin sialophosphoprotein (DSPP), dentin matrix protein 1 (DMP-1), and glyceraldehyde- 3-phosphate dehydrogenase (GAPDH) (Table 1). The amplification was carried out by a StepOne™ Real-Time PCR System (Applied Biosystems) with the following cycling conditions: cDNA denaturation and polymerase activation step at 95 °C for 10 min, followed by 40 cycles of denaturation at 95 °C for 15 s and annealing at 60 °C for 1 min. The relative gene expression analysis of target genes was performed in comparison with the GAPDH housekeeping control gene following the comparative 2 − ΔCt method. The normalized expression was calculated as fold change mRNA level versus control condition.

### 2.6. Statistical Analysis

The outcomes were shown as mean ± SEM of at least three independent replicates, as indicated for each experiment in the figure legends. Statistical analysis of the data was conducted using a one-way analysis of variance (one-way ANOVA), followed by Tukey’s test for multiple comparisons using GraphPad Prism 9.0 software (GraphPad Software, Inc., San Diego, CA, USA). A *p* lower than 0.05 was considered significant (* *p* < 0.05, ** *p* < 0.01, *** *p* < 0.001, **** *p* < 0.0001).

## 3. Results

### 3.1. Cell Viability

**Figure 1:**
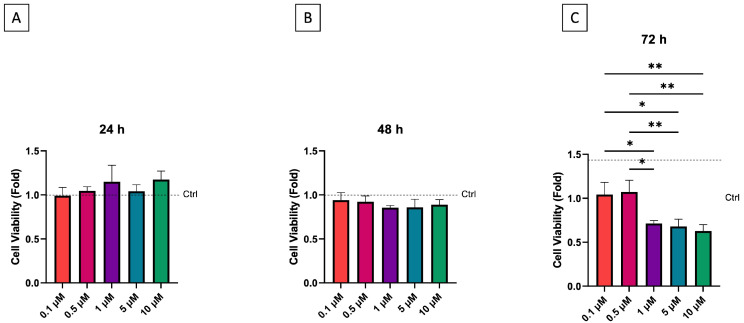
Cell viability in growth medium of simvastatin at concentration of 0.1, 0.5, 1, 5, and 10 µM evaluated with MTT ass after 24 (**A**), 48 (**B**), and 72 (**C**) hours (*n* = 3); * *p* < 0.05, ** *p* < 0.01.

To assess possible cytotoxicity on hDPSCs, cells were treated with simvastatin at concentrations of 0.1, 0.5, 1, 5, and 10 µM for 24, 48, and 72 h. As shown in Figure 1A, after 24 h of treatment, no significant reduction in cell viability was observed at any concentration compared to the untreated control group (ctrl) (0.1 µM, *p* > 0.999; 0.5 µM, *p* = 0.996; 1 µM, *p* = 0.628; 5 µM, *p* = 0.997; and 10 µM, *p* = 0.479). Moreover, no significant differences were observed between the various simvastatin concentrations. Similarly, after 48 h, all treated groups showed comparable viability to controls (0.1 µM, *p* = 0.948; 0.5 µM, *p* = 0.857; 1 µM, *p* = 0.345; 5 µM, *p* = 0.373; and 10 µM, *p* = 0.302), with no statistically significant differences observed among the tested concentrations (Figure 1B). On the other hand, after 72 h of treatment, higher concentrations of simvastatin significantly reduced hDPSC viability compared to the control group, specifically at 5 µM (*p* = 0.031) and 10 µM (*p* = 0.011). These concentrations decreased cell viability to approximately 0.3-fold relative to control, as shown in Figure 1C. Furthermore, cell viability in hDPSCs treated with 0.1 µM simvastatin was approximately 0.3-fold higher compared to those treated with 1 (*p* = 0.027), 5 (*p* = 0.014), and 10 µM (*p* = 0.005). Similarly, treatment with 0.5 µM simvastatin resulted in a 0.4-fold increase in viability relative to 1 (*p* = 0.005), 5 (*p* = 0.008), and 10 µM (*p* = 0.003).

### 3.2. Osteogenic Differentiation of hDPSCs

**Figure 2:**
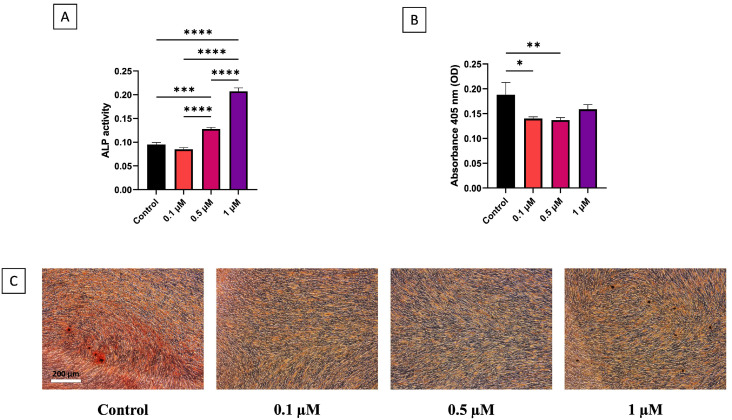
Alkaline Phosphatase activity depicted as representative enzymatic activity (*n* = 3) (**A**). Early mineralization evaluated by alizarin red representative quantization (**B**) and staining (**C**) (*n* = 3) * *p* < 0.05, ** *p* < 0.01, *** *p* < 0.001, **** *p* < 0.0001.

ALP is a well-established early marker of osteogenic differentiation; thus, its activity was evaluated in hDPSCs exposed to simvastatin at concentrations of 0.1, 0.5, and 1 µM. Cells cultured in differentiated medium were used as controls. The ALP activity trend was dose-dependent (Figure 2A); indeed, enzymatic activity increased proportionally with rising concentrations of simvastatin. Specifically, 1 µM showed the highest positive effect on ALP activity, showing a 0.1-fold enhancement in comparison to control and the other simvastatin concentrations (*p* = 0.0001). Furthermore, treatment with 0.5 µM simvastatin resulted in a 0.04 increase in ALP activity compared to control (*p* = 0.0002) and 0.1 µM simvastatin (*p* < 0.0001).

Detecting the capacity of hDPSCs to generate mineralization nodules, simvastatin 1 µM demonstrated a comparable influence regarding mineralization ability with respect to the control group. Furthermore, 0.1 µM did not seem to promote calcium deposits compared to control (*p* = 0.0105) and the same trend was observable in simvastatin 0.5 µM (*p* = 0.0073) (Figure 2B,C).

### 3.3. Odontogenic Gene Expression

**Figure 3:**
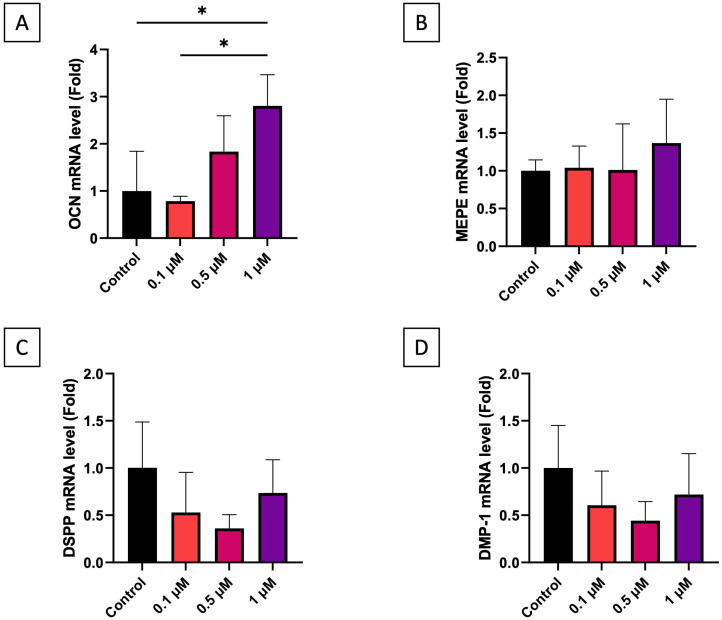
qPCR for odontogenic marker genes OCN (**A**), MEPE (**B**), DSPP (**C**), and DMP-1 (**D**) (*n* = 3 at least). * *p* < 0.05.

To analyze the odontoblast differentiation and resulting reparative dentinogenesis, the effects of simvastatin at concentrations of 0.1, 0.5, and 1 µM on the expression of mRNAs encoding OCN, MEPE, DSPP, and DMP-1 in hDPSCs were evaluated. In particular, the mRNA levels of OCN were significantly higher—by approximately 2-fold—in cells treated with simvastatin 1 µM than 0.1 µM (*p* = 0.0232) and control (*p* = 0.0231) (Figure 3A), while the expression levels of MEPE, DSPP, and DMP-1 did not differ among any of the simvastatin concentrations and the control group (Figure 3B–D).

## 4. Discussion

The long-term maintenance of dental elements has a significant impact on the correct development of oral apparatus, tooth function, and esthetic preservation. The chance to repair or even to regenerate dental hard and soft tissue provides a minimally invasive alternative that warrants significant consideration by clinicians as well as further investigations. Several materials have been proposed for VPT [24,25,26], and statins have shown their potential in favoring mineralized tissue formation [13,18,19,27,28]. Moreover, they were also found to promote the osteogenic differentiation of mesenchymal stem cells, which is considered a crucial process during dentin–pulp complex regeneration [13,18,19,27]. In this light, the aim of the current in vitro study was to evaluate the effect of simvastatin on the differentiation of hDPSCs into cells able to form a mineralized tissue, specifically assessing the expression of genes associated with dentinogenesis. The cytotoxicity of simvastatin was assessed across a range of concentrations, and the obtained results demonstrated that simvastatin at 1, 5, and 10 µM significantly reduced cell viability, consistently with a previous study on mesenchymal stem cells [29]. Consequently, subsequent differentiation assays were performed with non-cytotoxic concentrations (0.1 and 0.5 µM), along with 1 µM, as the lowest concentrations that negatively affected cell viability. Regarding cell differentiation, treatment with 1 µM simvastatin showed a positive influence on ALP activity along with OCN and MEPE expression, which was statistically significant in the first two. However, simvastatin had comparable (1 µM) and slightly less (0.1 and 0.5 µM) effect regarding early-stage mineralization ability than the control group, as well as no significant improvement in the expression of DSPP and DMP-1.

The significantly higher ALP activity in the presence of simvastatin was previously reported by Min et al. [27], who also described that the extent of mineralization was comparable to the control after 7 days and augmented at the 14-day evaluation. This might explain the similar 7-day early mineral deposition between test and control groups obtained in the present study, suggesting that a longer period of observation might provide improved mineralization with simvastatin treatment. Regarding differentiation markers, DSPP, DMP-1, OCN, and MEPE are specifically expressed by odontoblasts [25]. In particular, MEPE, an early phase differentiation marker [30], was upregulated following treatment with 1 µM simvastatin in hDPSCs, highlighting the potential contribution of statins in odontogenic differentiation. To the best of our knowledge, this is the first study evaluating MEPE expression in hDPSCs exposed to simvastatin. Similarly, OCN was significantly upregulated in the presence of simvastatin, in agreement with previous studies [13,27]. On the contrary, DSPP and DMP-1 did not show significant upregulation when exposed to simvastatin. Okamoto et al. [13] reported that after 7 days of culture, OCN was upregulated up to 18.1 times by simvastatin 1 µM compared to control and that DSPP was expressed up to 3.7 times. These quantitative differences in dentin-specific marker expression might support the results obtained in the current study. Moreover, Rewthamrongsris et al. reported a shift toward late-stage osteogenic differentiation stressed by the increased expression of Osterix (OSX), DMP1, DSPP, and OCN, along with a reduction in RUNX2 and ALP that correlated with mineral deposition results [31]. It should also be considered that the effect of statins on the regulation of inflammation and the promotion of angiogenesis was not considered. It has been demonstrated that simvastatin is associated with the expression of pro-inflammatory mediators and has a pro-angiogenic effect on endothelial cells [18,32]. These additional mechanisms might explain the limited upregulation of some dentin-specific markers, supporting the involvement of statins in different aspects of pulp healing. However, this role of statins was not evaluated by the present study and need to be further developed.

It has also been proposed that the presence of adjunctive materials—such as hydraulic cements—might contribute to ease cell differentiation [33,34,35,36]. It may be speculated that the combination of hydraulic cements and statins might synergically cause, on one hand, cell differentiation and the production of a mineralized matrix, and, on the other hand, decrease inflammation and promote angiogenesis, thereby enabling pulp tissue healing and repair [27,32], or, in specific cases, supporting the development of a pulp-like tissue [13,37]. Although the null hypothesis in the current study was partially rejected, previous research demonstrated that simvastatin positively affects the Wnt/β-catenin and BMP signaling pathways, promoting osteoblast differentiation through enhanced Runx2 activity and additionally supporting bone regeneration [38,39,40]. The main limitations of the present research are represented by the early-stage differentiation (7 days) and the absence of further analyses specifically related to odontoblastic differentiation potential (i.e., the detection of odontoblastic differentiation-related proteins). Further studies will be conducted to include additional time points, such as 14 and 21 days, to provide a more comprehensive understanding of the differentiation process over time. Moreover, the partial effect of simvastatin on dentin-specific marker expression stresses the need of additional studies focused on its role alone or in combination with other materials [41,42] in the regeneration of damaged dental pulp tissues and the promotion of reparative dentinogenesis.

## 5. Conclusions

Within the limitations of the present in vitro study, which primarily assessed early-stage differentiation, it could be concluded that simvastatin might demonstrate a positive influence in dentinogenesis, improving the expression of specific markers such as MEPE and OCN. However, its effect on inflammation reduction as well as its potential to be used in combination with other materials need further in-depth investigation to support its application in reparative and regenerative processes of the dentin–pulp complex.

## Figures and Tables

**Table 1:** T Primer sequences for qPCR analysis.

Primer	Forward Sequence (5′–3′)	Reverse Sequence (5′–3′)
OCN	CCACCGAGACACCATGAGAG	CCATAGGGCTGGGAGGTCAG
MEPE	GGTTATACAGATCTTCAAGAGAGAG	GTTGGTACTTTCAGCTGCATCACT
DSPP	AGAAGGACCTGGCCAAAAAT	TCTCCTCGGCTACTGCTGTT
DMP-1	TGGGGATTATCCTGTGCTCT	TACTTCTGGGGTCACTGTCG
GAPDH	TCAGCAATGCCTCCTGCAC	TCTGGGTGGCAGTGATGGC

## Data Availability

The data presented in this study are available on request from the corresponding author.

## Abbreviations

The following abbreviations are used in this manuscript:

hDPSCs	human Dental Pulp Stem Cells
ALP	Alkaline Phosphatase
ARS	Alizarin Red Staining
VPT	Vital Pulp Therapy
BMP	Bone Morphogenetic Protein
PCR	Polymerase Chain Reaction
OCN	Osteocalcin
MEPE	Matrix Extracellular Phosphoglycoprotein
DSPP	Dentin Sialophosphoprotein
DMP-1	Dentin Matrix Protein 1
GAPDH	Glyceraldehyde-3-Phosphate Dehydrogenase
OSX	Osterix
